# One Avocado per Day as Part of Usual Intake Improves Diet Quality: Exploratory Results from a Randomized Controlled Trial

**DOI:** 10.1016/j.cdnut.2024.102079

**Published:** 2024-01-11

**Authors:** Kristina S Petersen, Sydney Smith, Alice H Lichtenstein, Nirupa R Matthan, Zhaoping Li, Joan Sabate, Sujatha Rajaram, Gina Segovia-Siapco, David M Reboussin, Penny M Kris-Etherton

**Affiliations:** 1 Department of Nutritional Sciences, Pennsylvania State University, State College, PA, United States; 2 Department of Biostatistics and Data Science, Wake Forest University School of Medicine, Winston-Salem, NC, Unite States; 3 Jean Mayer USDA Human Nutrition Research Center on Aging, Tufts University, Medford, MA, United States; 4 Center for Human Nutrition, David Geffen School of Medicine at UCLA, Los Angeles, CA, United States; 5 Center for Nutrition, Healthy Lifestyle and Disease Prevention, School of Public Health, Loma Linda University, Loma Linda, CA, United States

**Keywords:** diet quality, Healthy Eating Index, cardiometabolic disease, avocado, randomized controlled trial, clinical trial, abdominal obesity, 24-hour recall, elevated waist circumference

## Abstract

**Background:**

Few clinical trials have evaluated diet quality change as a predictor of intervention effectiveness.

**Objectives:**

The aim of this study was to examine changes in the Healthy Eating Index (HEI)-2015 after a food-based intervention, and assess the associations between HEI-2015 change and intervention effects on cardiometabolic risk–related outcomes.

**Methods:**

The Habitual Diet and Avocado Trial was a 26-wk, multicenter, randomized, controlled parallel-arm study. Participants were 1008 individuals aged ≥25 y with abdominal obesity (females ≥ 35 inches; males ≥ 40 inches). The avocado-supplemented diet group was provided 1 avocado per day, and the habitual diet group maintained their usual diet. Change in diet quality was assessed using the HEI-2015 from a single 24-h recall conducted at 4 time points. Mixed models were used for analysis.

**Results:**

The avocado-supplemented diet group had a greater increase in the HEI-2015 (4.74 points; 95% CI: 2.93, 6.55) at 26 wk than the habitual diet group. Compared with the habitual diet group, the avocado-supplemented diet group had greater increases in the following HEI-2015 components from baseline: total vegetables (0.99 points; 95% CI: 0.77, 1.21), fatty acid ratio (2.25 points; 95% CI: 1.74, 2.77), sodium (1.03 points; 95% CI: 0.52, 1.55), refined grains (0.82 points; 95% CI: 0.32, 1.31), and added sugars (0.84 points; 95% CI: 0.49, 1.19). No differences in HEI-2015 improvements were observed by race, ethnicity, study site, body mass index, or age category. In the avocado-supplemented diet compared with the habitual diet group, the HEI-2015 increased in females (6.50 points; 95% CI: 4.39, 8.62) but not in males (0.02 points; 95% CI: −3.44, 3.48). Median HEI-2015 change was not associated with intervention-related changes in cardiometabolic disease risk factors.

**Conclusions:**

Intake of 1 avocado per day for 26 wk in adults with abdominal obesity increased adherence to the Dietary Guidelines for Americans. Changes in diet quality did not predict changes in risk factors for cardiometabolic disease.

This trial was registered at clinicaltrials.gov as NCT03528031 (https://clinicaltrials.gov/study/NCT03528031).

## Introduction

Poor diet quality is a leading preventable risk factor for cardiometabolic diseases [1], with ∼12% of cardiovascular disease deaths in the United States being attributed to poor diet quality [2]. Poor diet quality is defined by overconsumption of dietary components associated with increased risk of disease and low intakes of dietary components associated with reduced disease risk. Accordingly, diet quality indices are used to quantify the overall healthfulness of a dietary pattern based on its constituents [3]. This approach aligns with evidence showing that the totality of dietary exposures have a greater effect on health outcomes than individual dietary components [3]. The Healthy Eating Index (HEI)-2015 assesses adherence to the Dietary Guidelines for Americans and is a valid and reliable measure of diet quality [4]. The mean HEI-2015 score for United States individuals ≥2 y is 59 points of 100, with minimal change observed since 2005 [5]. Given that higher diet quality is strongly associated with lower risk of cardiometabolic diseases [[6], [7], [8], [9], [10]], which are leading contributors to US death and disability burden [11,12], strategies are needed to improve diet quality.

A systematic review of 25 dietary intervention studies including individuals at risk for cardiometabolic diseases showed clinically relevant (ie, 5- to 6-point increase on a 100-point scale) [13] increases in diet quality assessed by the HEI in several of the included studies [14]. These studies tended to examine multiple food behaviors or food-behavior goals and implemented intensive long-term interventions. Similarly in a systematic review of 18 trials examining behavioral weight loss interventions, improvements in HEI scores were typically in the range of 4–7 points [15]. However, it was noted that few conclusions could be drawn about the expected magnitude of HEI score change in response to a dietary intervention based on the studies conducted to date [14]. In part, this is because HEI is a multidimensional score that includes 9 adequacy components and 4 moderation components that are scored according to intake on a density (per 1000 kcal or percentage of total energy) basis of either a maximum of 5 or 10 points. Given this scoring system, combined with variability in baseline intake patterns, it can be difficult to predict the effect of intended dietary changes on diet quality. Moreover, the authors are not aware of any clinical trials that have evaluated relations between change in diet quality and changes in risk factors for cardiometabolic diseases.

With increased interest [16] and Federal support for “food is medicine” initiatives [17], there is a need to examine how food-based interventions alter diet quality and the relationship with risk factors for chronic diet-related diseases. We previously conducted a randomized controlled trial designed to test whether consuming 1 large avocado per day for 26 wk compared with habitual diet would decrease visceral adiposity in a diverse group of free-living individuals with abdominal obesity [18]. The findings demonstrated that the habitual dietary incorporation of 1 avocado per day did not reduce visceral adipose tissue volume and had minimal effect on risk factors associated with cardiometabolic disorders [18]. This ancillary study evaluated the magnitude of change in diet quality in response to a single food-based intervention and the association with changes in risk factors for cardiometabolic diseases. Based on data from the NHANES 2001–2008 [19] and 2001–2012 [20] showing adult avocado consumers have higher HEI-2005 scores (+6.3 and +9.3 points, respectively) than nonavocado consumers, it was hypothesized that improvements in diet quality would be observed. The aims of these exploratory analyses were as follows: *1*) to examine the change in HEI-2015 total and component scores by intervention group during the 26-wk study; *2*) to examine the consistency of HEI-2015 changes across subgroups within the cohort; *3*) to assess whether HEI-2015 change is associated with intervention effects on cardiometabolic disease risk factor outcomes.

## Methods

### Overview

The protocol and results of the primary and secondary outcomes have been reported previously [18,21]. This study presents the results of exploratory analyses examining changes in the HEI-2015 in response to the addition of 1 avocado a day over a 26-wk period. In addition, the associations between HEI-2015 change and intervention effects on cardiometabolic disease risk factors are assessed. In brief, the Habitual Diet and Avocado Trial (HAT) was a multicenter, randomized, controlled, parallel-arm, nonblinded study that included 1008 adults with abdominal obesity. The first participant was randomly assigned on 27 June 2018, and data collection was completed in October 2020. The avocado-supplemented diet group was provided with and asked to consume 1 avocado (∼168 g) per day for a nominal 26-wk period. Participants were provided with written instructions describing how to ripen, cut, remove the pit of, and peel avocados, in addition to serving ideas and recipes containing avocados. No additional dietary counseling or guidance was provided. The habitual diet group was asked to continue their usual diet and limit their avocado intake to ≤2 avocados per month. The study was reviewed by institutional review boards (IRBs) at each study site and then administered by a central IRB at the coordinating center (Wake Forest University IRB00047011). All participants provided written informed consent. The trial is registered at clinicaltrials.gov (identifier: NCT03528031).

### Participants

Participants were recruited from 4 study centers: Pennsylvania State University (with a site at University Park and a subsite at Hershey Medical Center), PA; Loma Linda University, CA; University of California at Los Angeles, CA; and Tufts University (Jean Mayer USDA Human Nutrition Research Center on Aging), Boston, MA. Inclusion criteria were age ≥25 y, waist circumference ≥35 inches for females and ≥40 inches for males, and low usual avocado intake (≤2 avocados per month). Key exclusion criteria were aversion to avocados, known avocado sensitivity, unwillingness to undergo magnetic resonance imaging scans (primary outcome assessment), unstable medical conditions (eg, dialysis for renal disease, cardiac, gastrointestinal, or hepatic disease, cancer (nonmelanoma skin cancer >5 y ago acceptable, any cancer site >10 y without recurrence), unstable weight history (lost or gain of >4.5 kg in past 12 mo), following a restrictive or weight loss diet, and habitual alcohol intake ≥7 drinks per week for females and ≥14 drinks per week for males.

### Outcome variable assessments

The primary outcome for which this study was powered was visceral adipose tissue volume; sample size calculations are reported in the protocol article [21]. As previously reported [21] visceral adipose tissue volume and hepatic fat fraction were measured by magnetic resonance imaging at the beginning and end of the intervention period. Height, body weight and waist circumference, and fasting blood samples were obtained at baseline, 12 wk, and 26 wk. Blood samples were analyzed for C-reactive protein, lipids/lipoproteins, glucose, and insulin at the Central Laboratory, as previously described [21].

### Diet assessment and HEI-2015 calculation

Unannounced 24-h recalls were completed at 4 time points during the trial. A single recall was completed before randomization (ie, baseline), and within 1–2 wk of study week 8, 16, and 26. The 24-h recalls were conducted by telephone using the Nutrition Data System for Research (NDSR) versions 2017 and 2018. The baseline 24-h recall was completed on any day of the week. For the postrandomization 24-h recalls, the goal was to obtain 2 weekday and 1 weekend day recalls across the 3 time points (ie, week 8, 16, and 26). Using a standardized manual of procedures for collection and management of dietary data, 2 diet assessment sites were each responsible for collecting 50% of the 24-h recalls across all intervention sites.

The HEI-2015 was calculated using the simple HEI-2015 scoring algorithm method according to SAS code downloaded from the Nutrition Coordinating Center website [22]. It should be noted that the recently released HEI-2020 is exactly the same as the HEI-2015, although the index was renamed to reflect the alignment with the 2020–2025 Dietary Guidelines for Americans [23]. HEI-2015 scores were calculated for each intake day for every participant. Previously, it has been demonstrated that calculation of the HEI-2005 with NDSR based on the method developed for use with the USDA Food and Nutrient Dietary Data System and MyPyramid Equivalents Databases yields similar HEI-2005 scores to HEI-2005 calculations based on the Food and Nutrient Dietary Data System and MyPyramid Equivalents Databases [24]. However, NDSR classifies avocados differently than the USDA databases. NDSR classifies avocados as a fruit, and therefore, avocado intake is attributed to the total and whole fruit HEI-2015 components, whereas, in the USDA databases, avocados are classified as a vegetable and attributed to the total vegetable HEI-2015 component. Given the focus of this clinical trial and our objective of determining the effect of increasing avocado intake on adherence to the Dietary Guidelines for Americans, we modified the NDSR HEI-2015 calculation to align with the USDA classification of avocados as a vegetable. Specifically, avocado servings were included in the total vegetable HEI-2015 component and removed from the HEI-2015 fruit components. The NDSR avocado serving size was not modified (½ cup chopped; ∼75 g).

### Statistical analyses

Descriptive tables (means and SDs) for the total HEI-2015 score and HEI-2015 component scores were calculated by intervention group and study week and by participant subgroups. Descriptive statistics (counts and percentages) were calculated for avocado consumption by intervention group and study week. Linear mixed models adjusting for repeated measures with an unstructured covariance matrix were used to estimate the between-group difference in the change in the total HEI-2015 score and HEI-2015 component scores from baseline and to examine the consistency of HEI-2015 changes across subgroups within the cohort. The models were adjusted for site and included the interactive effect of intervention group and visit. Sensitivity analyses were performed by adjusting for baseline total HEI-2015 score. The total HEI-2015 baseline score covariate was constructed by categorizing HEI-2015 scores at baseline into quartiles (quartile 1: <45 points; quartile 2: 45–54 points; quartile 3: 55–64 points; quartile 4: ≥65 points). The baseline score covariate for each of the HEI-2015 components was constructed by dividing the range of the component score into 4 equal intervals (ie, for components worth 5 points the categories were 0, >0 and ≤2.5, >2.5 and <5, 5 points; for components worth 10 points the categories were 0, >0 and ≤5, >5 and ≤10, 10 points). Between-group differences in the change in total HEI-2015 score by subgroups were tested by adding the subgroup of interest as a categorical covariate to the mixed effect models. The *P* values from the interaction term of subgroup and intervention group were used to test the significance of subgroup effects. Subgroup analyses were conducted by sex, ethnicity, race, BMI category, age category, study site, and baseline HEI-2015 score.

Mixed models were used to assess the association between HEI-2015 change and intervention effects on cardiometabolic disease risk factors. HEI-2015 change was modeled as the median change in total HEI-2015 score from baseline to all follow-up total scores. First, we calculated the median change in total HEI-2015 score from baseline for each participant and then categorized the median change into 4 levels (decrease >−5 points; no change −5 to + 5 points; increase > 5–10 points; increase >10 points). For outcomes with multiple postrandomization assessments, mixed models were used to estimate the between-group difference in the change in the outcome by categorized change in median total HEI-2015 score adjusted for intervention group and site. For outcomes with a single follow-up measure, linear regression models adjusted for intervention group and site were used to estimate the between-group difference in the 26-wk change from baseline by categorized change in median total HEI-2015 score. For both types of analyses, an interaction term of categorized change in median total HEI-2015 by intervention group was included. The significance of the interaction term was used to test the effect of change in total HEI-2015 score from baseline on the change in outcomes from baseline. Analyses were conducted consistent with intention-to-treat principles and included all available data. Normality was assessed for the residuals of the HEI-2015 total and component scores using residual plots produced from model outputs. Formal tests of normality were not necessary because of the large sample size. Analyses were performed with SAS 9.4 (SAS Institute), with *P* values presented as nominal values and not adjusted for multiple comparisons. *P* values were considered statistically significant at *α* < 0.05

## Results

The characteristics of the study cohort and participant flow through the study have been previously described [18]. In brief, the sample included 1008 randomly assigned participants (72% females) with a mean age of 50 ± 14 y and a mean BMI of 33.0 ± 5.5 kg/m^2^. The mean waist circumference of females and males was 106 ± 12 cm and 118 ± 12 cm, respectively. The self-reported racial and ethnic distribution of the cohort was 69% White, 21% Hispanic, 15% Black, 6% Asian, and 10% either did not answer or were American Indian, other, or multiple races or ethnicities.

### Change in the HEI-2015

The total HEI-2015 and component scores are presented by intervention arm at baseline and during the trial in Table 1. At 26 wk, a greater increase in the HEI-2015 score was observed in the avocado-supplemented diet group than in the habitual diet group (4.74 points; 95% CI: 2.93, 6.55) (Table 2). After adjustment for baseline HEI-2015 score, the magnitude of the effect was slightly attenuated (4.34 points; 95% CI: 3.03, 5.65) (Supplemental Table 1). The increases in HEI-2015 observed in the avocado-supplemented diet group were driven by higher scores for the total vegetables, fatty acid ratio, sodium, refined grains, and added sugars components. Small reductions in the total protein foods and seafood and plant protein components were observed in the avocado-supplemented diet group compared with the habitual diet group. Similar component score results were observed after baseline adjustment, except for the dairy component. After baseline adjustment, the dairy component score was lower in the avocado-supplemented diet group compared with the habitual diet group.

**TABLE 1:** HEI-2015 total and component scores by intervention group at baseline and during the 26 wk of HAT (*n* = 1008)^1^

Variables	Avocado-supplemented diet group				Habitual diet group			
	Baseline	Wk 8	Wk 16	Wk 26	Baseline	Wk 8	Wk 16	Wk 26
	*n* = 505	*n* = 490	*n* = 479	*n* = 472	*n* = 503	*n* = 486	*n* = 487	*n* = 480
HEI-2015 score^2^	53.3 (15.1)	58.6 (13.7)	58.0 (13.2)	58.2 (13.5)	54.0 (14.5)	53.8 (14.8)	53.9 (15.1)	54.7 (15.4)
Total vegetables^3^	3.4 (1.7)	4.4 (1.0)	4.4 (1.0)	4.3 (1.1)	3.4 (1.7)	3.3 (1.7)	3.3 (1.7)	3.4 (1.6)
Greens and beans^3^	2.3 (2.3)	2.2 (2.3)	2.2 (2.3)	2.2 (2.3)	2.2 (2.3)	2.4 (2.3)	2.3 (2.3)	2.3 (2.3)
Total fruit^3^	1.7 (1.9)	1.6 (1.8)	1.7 (1.9)	1.7 (1.9)	1.8 (2.0)	1.8 (2.0)	1.8 (2.0)	1.8 (1.9)
Whole fruit^3^	2.0 (2.2)	1.9 (2.2)	1.9 (2.2)	2.0 (2.2)	2.0 (2.2)	2.0 (2.2)	2.1 (2.2)	2.1 (2.2)
Whole grains^4^	3.8 (4.1)	3.9 (4.0)	3.7 (3.9)	3.9 (4.1)	4.0 (4.1)	3.9 (4.1)	3.9 (4.2)	4.0 (4.0)
Dairy^4^	4.7 (3.4)	4.3 (3.3)	4.6 (3.3)	4.2 (3.2)	4.7 (3.5)	4.8 (3.6)	4.8 (3.5)	4.8 (3.5)
Total protein foods^3^	4.3 (1.3)	4.1 (1.4)	4.1 (1.4)	4.2 (1.4)	4.4 (1.2)	4.5 (1.1)	4.4 (1.2)	4.4 (1.2)
Seafood and plant protein^3^	2.6 (2.3)	2.3 (2.3)	2.4 (2.3)	2.2 (2.4)	2.5 (2.4)	2.6 (2.4)	2.5 (2.3)	2.7 (2.4)
Fatty acid ratio^4^	5.0 (3.8)	7.4 (3.2)	7.1 (3.2)	7.3 (3.2)	5.0 (3.8)	4.9 (3.8)	5.0 (3.8)	5.0 (3.8)
Sodium^4^	4.4 (3.8)	5.7 (3.7)	5.2 (3.8)	5.6 (3.8)	4.4 (3.7)	4.3 (3.8)	4.4 (3.8)	4.7 (3.8)
Refined grains^4^	6.3 (3.8)	7.2 (3.5)	7.1 (3.5)	7.1 (3.5)	6.5 (3.8)	6.6 (3.8)	6.5 (3.8)	6.6 (3.8)
Added sugars^4^	7.5 (2.9)	8.3 (2.6)	8.5 (2.3)	8.3 (2.5)	8.0 (2.7)	7.9 (2.7)	7.9 (2.8)	8.1 (2.5)
Saturated fats^4^	5.3 (3.5)	5.4 (3.6)	5.1 (3.6)	5.2 (3.5)	5.0 (3.6)	5.0 (3.7)	5.0 (3.7)	4.9 (3.7)

1 Data presented as mean (SD); Abbreviations: HAT, Habitual Diet and Avocado Trial; HEI, Healthy Eating Index.

2 Maximum score 100.

3 Maximum score 5.

4 Maximum score 10.

**TABLE 2:** Model-based estimates of changes in HEI-2015 total and component scores by intervention group during the 26 wk of HAT (*n* = 1008)^1^

Variable	Avocado-supplemented diet group			Habitual diet group			Estimated between-group difference	
	Wk 8, *n* = 490	Wk 16, *n* = 479	Wk 26, *n* = 472	Wk 8, *n* = 486	Wk 16, *n* = 487	Wk 26, *n* = 480	Mean (95% CI)	*P*
HEI-2015 score^2^	5.03	4.39	4.52	−0.40	−0.32	0.44	4.74 (2.93, 6.55)	<0.001
Total vegetables^3^	0.97	0.94	0.89	−0.06	−0.14	0.01	0.99 (0.77, 1.21)	<0.001
Greens and beans^3^	−0.11	−0.16	−0.14	0.21	0.16	0.11	−0.30 (−0.62, 0.02)	0.07
Total fruit^3^	−0.17	−0.04	−0.05	−0.09	−0.02	−0.04	−0.04 (−0.28, 0.21)	0.78
Whole fruit^3^	−0.11	−0.06	0.05	−0.02	0.09	0.10	−0.10 (−0.38, 0.19)	0.51
Whole grains^3^	0.04	−0.17	0.01	−0.23	−0.22	−0.11	0.15 (−0.40, 0.69)	0.60
Dairy^4^	−0.35	−0.06	−0.45	0.04	0.07	0.09	−0.35 (−0.81, 0.10)	0.13
Total protein foods^3^	−0.21	−0.19	−0.14	0.09	0.02	0.02	−0.22 (−0.39, −0.05)	0.01
Seafood and plant protein^3^	−0.24	−0.20	−0.43	0.05	0.01	0.14	−0.36 (−0.66, −0.05)	0.02
Fatty acid ratio^4^	2.25	2.04	2.18	−0.10	−0.08	−0.10	2.25 (1.74, 2.77)	<0.001
Sodium^4^	1.20	0.70	1.06	−0.23	−0.11	0.21	1.03 (0.52, 1.55)	<0.001
Refined grains^4^	0.90	0.83	0.84	0.07	−0.04	0.08	0.82 (0.32, 1.31)	0.001
Added sugars^4^	0.75	0.85	0.70	−0.13	−0.11	0.02	0.84 (0.49, 1.19)	<0.001
Saturated fats^4^	0.11	−0.11	−0.002	−0.01	0.04	−0.10	0.02 (−0.46, 0.50)	0.94

Abbreviations: HAT, Habitual Diet and Avocado Trial; HEI, Healthy Eating Index.

1 Mixed models were used to estimate the between-group difference in the change in total HEI-2015 score and HEI-2015 component scores from baseline with adjustment for study site.

2 Maximum score 100.

3 Maximum score 5.

4 Maximum score 10.

### Change in the HEI-2015 by subgroups

The total HEI-2015 and component scores were similar when assessed by intervention arm across the different participant subgroups (Table 3). Subgroup analyses showed no differences in HEI-2015 change by race, ethnicity, BMI category, age group, or study site (Table 4). The HEI-2015 effect differed by sex (*P* = 0.002). In the avocado-supplemented diet group compared with the habitual diet group, the HEI-2015 score increased in females (6.50 points; 95% CI: 4.39, 8.62) but not in men (0.02 points; 95% CI: −3.44, 3.48). However, this observation was attenuated (*P* = 0.16) after adjustment for baseline total HEI-2015 score, suggesting the effect was driven by differences in baseline HEI-2015 scores (Supplemental Table 2).

**TABLE 3:** Total HEI-2015 by intervention group and subgroup at baseline and during the 26 wk of HAT (*n* = 1008)

HEI-2015 score	Avocado-supplemented diet group				Habitual diet group			
	Baseline	Wk 8	Wk 16	Wk 26	Baseline	Wk 8	Wk 16	Wk 26
	*n* = 505	*n* = 490	*n* = 479	*n* = 472	*n* = 503	*n* = 486	*n* = 487	*n* = 480
Sex								
Female	53.0 (15.4)	59.4 (13.6)	58.1 (13.6)	58.8 (13.5)	55.3 (14.2)	54.5 (14.7)	54.5 (14.9)	55.0 (15.7)
Male	54.0 (14.4)	56.7 (13.9)	57.8 (12.3)	56.7 (13.3)	50.1 (14.6)	51.8 (14.9)	52.1 (15.6)	53.9 (14.6)
Self-reported ethnicity								
Not Hispanic/Latino	53.7 (14.9)	58.1 (13.9)	58.4 (13.0)	58.5 (13.7)	53.9 (15.0)	53.6 (14.5)	53.6 (15.1)	54.4 (15.5)
Hispanic/Latino	51.6 (15.6)	60.6 (13.0)	56.7 (14.2)	56.9 (12.8)	54.6 (12.2)	54.9 (15.5)	55.0 (15.3)	56.1 (15.2)
Self-reported race								
Black	52.4 (15.2)	58.4 (14.1)	60.1 (13.9)	59.2 (12.1)	51.9 (14.9)	51.9 (15.1)	51.5 (15.7)	54.1 (16.4)
Asian	55.3 (14.9)	62.3 (12.0)	61.0 (12.1)	57.7 (12.3)	52.0 (15.1)	54.5 (13.9)	53.0 (15.5)	52.3 (15.6)
White	53.2 (15.0)	58.3 (13.7)	57.5 (13.1)	58.1 (13.6)	54.4 (14.6)	53.7 (14.8)	54.3 (15.0)	54.7 (15.5)
Other	53.9 (15.9)	59.2 (15.0)	57.7 (14.0)	58.0 (15.5)	55.7 (12.7)	56.3 (14.5)	55.3 (14.2)	57.1 (13.3)
BMI								
Healthy/overweight	55.8 (15.9)	58.0 (14.1)	60.6 (12.3)	59.3 (13.9)	56.9 (15.3)	57.2 (15.2)	56.1 (14.3)	57.3 (15.7)
Obesity class I	53.0 (15.4)	59.8 (13.9)	57.5 (13.7)	58.7 (13.3)	53.4 (13.9)	52.6 (14.2)	53.4 (16.6)	54.8 (15.3)
Obesity class II	51.7 (13.4)	58.3 (13.3)	56.1 (13.5)	56.3 (13.7)	51.2 (13.9)	53.8 (14.3)	52.8 (13.9)	52.8 (15.9)
Obesity class III	49.6 (12.8)	56.6 (12.7)	55.4 (12.6)	56.1 (12.5)	52.4 (13.9)	48.0 (13.5)	51.0 (13.1)	51.0 (13.4)
Age group (y)								
25–30	56.3 (17.1)	60.4 (14.6)	57.2 (15.7)	59.3 (13.5)	52.6 (13.6)	55.4 (13.4)	52.5 (14.2)	55.9 (13.5)
31–59	51.2 (14.3)	57.5 (13.7)	57.2 (12.7)	57.1 (13.0)	52.6 (14.1)	52.3 (14.7)	52.4 (15.4)	53.1 (15.3)
60+	57.4 (15.2)	60.7 (13.3)	60.3 (13.3)	60.3 (14.5)	57.7 (15.1)	56.9 (14.9)	57.8 (14.0)	58.0 (15.8)
Site								
LLU	50.6 (14.8)	59.6 (13.3)	55.7 (13.7)	59.1 (14.5)	55.2 (12.4)	53.0 (14.3)	54.4 (15.3)	53.5 (14.8)
UCLA	54.6 (14.8)	60.0 (14.0)	59.9 (12.3)	58.7 (12.8)	55.1 (15.6)	55 (15.1)	55.8 (16.0)	57.9 (16.0)
Penn State—UP	52.4 (16.2)	54.4 (13.0)	56.4 (12.1)	55.4 (13.0)	52.6 (14.6)	53.5 (13.7)	51.6 (13.9)	52.8 (15.7)
Penn State—Hershey	53.9 (12.9)	59.0 (14.2)	56.4 (13.9)	56.8 (12.7)	54.5 (14.0)	55.6 (13.9)	56.6 (15.1)	56.1 (14.4)
Tufts	54.7 (15.8)	58.5 (13.8)	60.1 (13.4)	59.0 (13.7)	52.1 (15.4)	52.8 (15.8)	51.5 (14.1)	53.3 (15.4)
Baseline HEI-2015 score								
<45 points	36.3 (6.3)	55.9 (13.0)	55.5 (13.3)	54.5 (11.6)	37.4 (6.2)	47.3 (13.5)	47.8 (14.2)	48.7 (13.5)
45–54 points	49.7 (2.9)	54.7 (13.2)	56.7 (12.6)	58.4 (12.6)	49.8 (3.0)	52.1 (14.1)	53.8 (15.4)	53.6 (14.6)
55–64 points	59.7 (2.7)	61.9 (13.3)	58.6 (12.9)	57.9 (13.3)	59.6 (2.6)	56.3 (13.9)	56.0 (13.3)	55.2 (14.1)
65+ points	74.0 (6.9)	63.1 (13.9)	62.1 (13.2)	63.2 (15.4)	73.0 (7.0)	61.1 (13.7)	59.2 (14.7)	62.2 (16.2)

Data presented as mean (SD).

Abbreviations: BMI, body mass index; HAT, Habitual Diet and Avocado Trial; HEI, Healthy Eating Index; LLU, Loma Linda University; Penn State—UP, Penn State University—University Park; Penn State—Hershey, Penn State College of Medicine, Hershey; Tufts, Tufts University; UCLA, University of California at Los Angeles.

**TABLE 4:** Model-based estimates of the change in total HEI-2015 in subgroups by intervention group at 26 wk in HAT^1^

Subgroups	*n*	Avocado-supplemented diet group mean change	Habitual diet group mean change	Estimated between-group difference in change			
				Mean	*P*	95% CI	*P*^2^
Sex							0.002
Female	730	5.56	−0.94	6.50	<0.001	4.39, 8.62	
Male	278	2.38	2.36	0.02	>0.99	−3.44, 3.48	
Self-reported ethnicity							0.72
Not Hispanic or Latino	797	4.34	−0.27	4.61	<0.001	2.58, 6.64	
Hispanic or Latino	207	5.97	0.53	5.44	0.008	1.43, 9.44	
Self-reported race							0.93
Black	149	6.05	0.46	5.60	0.02	0.86, 10.34	
Asian	58	5.09	1.00	4.09	0.29	−3.42, 11.61	
White	694	4.55	−0.45	5.00	<0.001	2.81, 7.18	
Other	104	3.58	0.28	3.31	0.26	−2.41, 9.03	
BMI							0.39
Healthy/overweight	317	3.24	−0.25	3.48	0.03	0.27, 6.70	
Obesity class I	386	5.51	−0.28	5.79	0.0001	2.85, 8.72	
Obesity class II	192	5.02	1.98	3.04	0.15	−1.12, 7.19	
Obesity class III	112	4.80	−3.18	7.99	0.005	2.47, 13.51	
Age group (y)							0.14
25–30	96	2.19	1.92	0.27	0.93	−5.73, 6.27	
31–59	644	5.65	−0.34	5.99	<0.001	3.72, 8.25	
60+	268	3.02	−0.26	3.28	0.06	−0.19, 6.75	
Site							0.10
LLU	251	7.39	−1.59	8.98	<0.001	5.39, 12.57	
UCLA	251	4.68	0.98	3.69	0.046	0.06, 7.33	
Penn State—UP	134	2.25	−0.03	2.28	0.37	−2.66, 7.22	
Penn State—Hershey	119	3.51	1.48	2.04	0.45	−3.22, 7.29	
Tufts	253	4.10	0.04	4.07	0.03	0.46, 7.67	

Abbreviations: BMI, body mass index; HAT, Habitual Diet and Avocado Trial; HEI, Healthy Eating Index; LLU, Loma Linda University; Penn State—UP, Penn State University—University Park Campus; Penn State—Hershey, Penn State College of Medicine, Hershey; Tufts, Tufts University; UCLA, University of California at Los Angeles.

1 Mixed models were used to estimate the between-group difference in the change in total HEI-2015 score from baseline by subgroup with adjustment for study site. The *P* values from the interaction term of subgroup and intervention group were used to test the significance of subgroup effects.

2 *P* value for difference between intervention estimates within a subgroup.

More than 76% of the participants in the avocado-supplemented diet group reported consuming 1 avocado per day throughout the trial and an additional 12% of participants reported some avocado intake (>0 and <90% of the study-provided amount of 168 g) (Supplemental Table 3). More than 91% of participants in the habitual diet group reported no avocado consumption during the trial. When the change in the HEI-2015 was examined for the avocado-supplemented diet group by reported avocado intake level, greater increases in the HEI-2015 were observed with better adherence to the intervention. Compared with participants in the avocado-supplemented diet group who consumed no avocado, those who consumed some (7.24 points; 95% CI: 3.90, 10.58) or ≥1 avocado per day (7.17 points; 95% CI: 4.34, 10.01) had significantly higher HEI-2015 scores with no difference by intake level.

### Association between HEI-2015 change and intervention effects on cardiometabolic disease risk factors

No intervention or intervention by median HEI-2015 interactions were observed for visceral adipose tissue volume, hepatic fat fraction, C-reactive protein, metabolic syndrome criteria and body weight, BMI, insulin, very low-density lipoprotein cholesterol, and the total cholesterol to HDL cholesterol ratio (Table 5). The avocado-supplemented diet group had lower total cholesterol and LDL cholesterol concentrations compared to the habitual diet group; however, there was no intervention by median HEI-2015 score interaction (Table 6). In exploratory analyses, LDL cholesterol concentration lowering was greater in the avocado-supplemented diet group, compared to the habitual diet group, when the median change in HEI-2015 was > 10 points (−5.36 mg/dL; 95% CI: −9.66, −1.06).

**TABLE 5:** Model-based estimates of the change in primary, secondary, and metabolic syndrome-related outcomes by median HEI-2015 change category by intervention group at 26 wk in HAT

Outcomes	*n*	Avocado-supplemented diet group mean change	Habitual diet group mean change	Estimated between-group difference in change		
				Mean	95% CI	*P*
Primary outcome						
Visceral adipose tissue^1^ (L)						
All participants		**0.07**	**0.06**	**0.02**	−**0.02, 0.06**	**0.41**
Median ΔHEI-2015						0.13^2^
Decrease >5 points	293	0.12	0.08	0.04	−0.04, 0.11	0.30
No change	239	0.04	0.08	−0.05	−0.13, 0.03	0.23
Increase 5–10 points	105	0.05	0.06	−0.01	−0.13, 0.11	0.90
Increase >10 points	284	0.07	−0.01	0.08	0.00, 0.16	0.04
Secondary outcomes						
Hepatic fat fraction^1^ (%)						
All participants		**0.58**	**0.21**	**0.37**	−**0.31, 1.06**	**0.29**
Median ΔHEI-2015						0.45^2^
Decrease >5 points	295	1.48	0.59	0.89	−0.33, 2.11	0.15
No change	240	0.13	0.55	−0.41	−1.76, 0.94	0.55
Increase 5–10 points	104	0.59	−0.63	1.21	−0.83, 3.25	0.24
Increase >10 points	283	0.16	−0.36	0.52	−0.75, 1.78	0.42
CRP^3^ (mg/L)						
All participants		**0.79**	**0.44**	**0.35**	−**0.85, 1.55**	**0.56**
Median ΔHEI-2015						0.83^2^
Decrease >5 points	292	1.51	0.89	0.62	−1.58, 2.81	0.58
No change	259	1.21	0.04	1.18	−1.15, 3.51	0.32
Increase 5–10 points	109	0.04	0.57	−0.53	−4.10, 3.03	0.77
Increase >10 points	303	0.10	0.17	−0.06	−2.26, 2.13	0.95
Components of metabolic syndrome						
Waist circumference^3^ (cm)						
All participants		**0.01**	−**0.11**	**0.12**	−**0.32, 0.55**	**0.60**
Median ΔHEI-2015						0.63^2^
Decrease >5 points	299	0.31	0.37	−0.06	−0.85, 0.73	0.88
No change	259	−0.18	−0.41	0.23	−0.61, 1.08	0.59
Increase 5–10 points	111	−0.24	0.08	−0.30	−1.59, 0.99	0.65
Increase >10 points	303	−0.02	−0.57	0.55	−0.26, 1.36	0.18
Systolic BP^3^ (mm Hg)						
All participants		−**1.29**	−**0.03**	−**1.25**	−**2.57, 0.06**	**0.06**
Median ΔHEI-2015						0.26^2^
Decrease >5 points	306	−1.34	0.72	−2.06	−4.21, 0.09	0.06
No change	261	−1.41	0.56	−1.97	−4.27, 0.33	0.09
Increase 5–10 points	112	−0.14	−0.12	−0.02	−3.52, 3.47	0.99
Increase >10 points	306	0.07	−0.54	0.62	−1.56, 2.79	0.58
Diastolic BP^3^ (mm Hg)						
All participants		−**1.01**	−**0.32**	−**0.69**	−**1.60, 0.23**	**0.14**
Median ΔHEI-2015						0.50^2^
Decrease >5 points	306	−1.34	0.09	−1.42	−2.88, 0.03	0.06
No change	261	−1.02	−0.89	−0.15	−1.70, 1.40	0.85
Increase 5–10 points	112	−0.56	−0.92	0.36	−2.00, 2.72	0.76
Increase >10 points	306	−1.12	−0.85	−0.27	−1.74, 1.20	0.72
Triglyceride^3^ (mg/dL)						
All participants		**2.96**	**4.07**	−**1.11**	−**7.62, 5.40**	**0.74**
Median ΔHEI-2015						0.93^2^
Decrease >5 points	299	2.11	2.17	−0.05	−11.93, 11.83	0.99
No change	259	2.43	5.60	−3.17	−15.92, 9.59	0.63
Increase 5–10 points	111	−5.62	1.06	−6.68	−26.03, 12.67	0.50
Increase >10 points	302	6.67	6.84	−0.17	−12.21, 11.87	0.98
Glucose^3^ (mg/dL)						
All participants		**0.63**	**1.58**	−**0.95**	−**2.96, 1.07**	**0.36**
Median ΔHEI-2015						0.45^2^
Decrease >5 points	299	1.69	0.81	0.88	−2.80, 4.55	0.64
No change	259	0.06	2.71	−2.65	−6.59, 1.29	0.19
Increase 5–10 points	111	−1.16	2.49	−3.66	−9.63, 2.32	0.23
Increase >10 points	302	0.75	0.91	−0.16	−3.88, 3.56	0.93
HDL-C^3^ (mg/dL)						
All participants		−**0.28**	−**0.18**	−**0.10**	−**0.89, 0.69**	**0.81**
Median ΔHEI-2015						0.24^2^
Decrease >5 points	295	−0.18	0.02	−0.20	−1.64, 1.24	0.79
No change	258	−0.50	0.61	−1.10	−2.63, 0.44	0.16
Increase 5–10 points	110	0.71	−0.79	1.50	−0.84, 3.84	0.21
Increase >10 points	301	−0.53	−1.10	0.58	−0.88, 2.03	0.44

Abbreviations: BP, blood pressure; HAT, Habitual Diet and Avocado Trial; HDL-C, high-density lipoprotein cholesterol; HEI, Healthy Eating Index; CRP, C-reactive protein.

1 Linear regression models adjusted for intervention arm and site were used to estimate the association between HEI-2015 change and intervention effects on cardiometabolic risk factors and make comparisons between intervention groups. The categorized change in the median total HEI-2015 score was included in the model along with an interaction term by intervention group.

2 *P* value is for the interaction term (median ΔHEI-2015 × intervention group).

3 Mixed models were used to assess the association between HEI-2015 change and intervention effects on cardiometabolic risk factors and make comparisons between intervention groups. The categorized change in the median total HEI-2015 score was included in the model along with an interaction term by intervention group.

**TABLE 6:** Model-based estimates of the change in additional and post hoc outcomes by median HEI-2015 change category by intervention group at 26 wk in HAT^1^

Outcomes	*n*	Avocado-supplemented diet group	Habitual diet group	Estimated between-group difference in change		
				Mean	95% CI	*P*
Additional and post hoc outcomes						
Body weight (kg)						
All participants		**0.38**	**0.38**	**0.00**	−**0.31, 0.32**	**0.98**
Median ΔHEI-2015						0.35^2^
Decrease >5 points	299	0.62	0.56	0.06	−0.52, 0.63	0.85
No change	260	0.42	0.69	−0.26	−0.87, 0.35	0.40
Increase 5–10 points	111	0.25	0.34	−0.09	−1.02, 0.84	0.85
Increase >10 points	302	0.23	−0.27	0.49	−0.09, 1.07	0.10
BMI (kg/m^2^)						
All participants		**0.14**	**0.13**	**0.01**	−**0.10, 0.12**	**0.88**
Median ΔHEI-2015						0.39^2^
Decrease >5 points	299	0.23	0.20	0.04	−0.17, 0.24	0.73
No change	260	0.15	0.23	−0.09	−0.30, 0.13	0.44
Increase 5–10 points	111	0.11	0.13	−0.02	−0.35, 0.31	0.89
Increase >10 points	302	0.09	−0.09	0.17	−0.03, 0.38	0.10
Insulin (μIU/mL)						
All participants		**0.12**	**0.62**	−**0.50**	−**2.09, 1.10**	**0.54**
Median ΔHEI-2015						0.46^2^
Decrease >5 points	296	0.15	0.43	−0.28	−3.18, 2.63	0.85
No change	258	−2.26	0.53	−2.79	−5.91, 0.32	0.08
Increase 5–10 points	108	1.09	0.13	0.96	−3.80, 5.73	0.69
Increase >10 points	301	1.43	1.33	0.10	−2.83, 3.03	0.94
Total cholesterol (mg/dL)						
All participants		−**4.91**	−**1.97**	−**2.94**	−**5.54,** −**0.35**	**0.03**
Median ΔHEI-2015						0.62^2^
Decrease >5 points	299	−5.38	−1.11	−4.27	−9.01, 0.47	0.08
No change	259	−4.56	−3.26	−1.30	−6.38, 3.78	0.62
Increase 5–10 points	111	−3.31	−3.61	0.31	−7.40, 8.02	0.94
Increase >10 points	302	−5.36	−0.92	−4.43	−9.23, 0.37	0.07
LDL-C (mg/dL)						
All participants		−**4.95**	−**2.48**	−**2.47**	−**4.80,** −**0.13**	**0.04**
Median ΔHEI-2015						0.15^2^
Decrease >5 points	295	−5.49	−1.48	−4.01	−8.27, 0.26	0.07
No change	258	−4.05	−4.93	0.88	−3.66, 5.42	0.70
Increase 5–10 points	110	−2.45	−3.58	1.13	−5.80, 8.05	0.75
Increase >10 points	301	−5.97	−0.62	−5.36	−9.66, −1.06	0.01
VLDL-C (mg/dL)						
All participants		**0.59**	**0.81**	−**0.22**	−**1.53, 1.08**	**0.74**
Median ΔHEI-2015						0.93^2^
Decrease >5 points	299	0.42	0.43	−0.01	−2.39, 2.37	0.99
No change	259	0.49	1.12	−0.63	−3.19, 1.92	0.63
Increase 5–10 points	111	−1.12	0.21	−1.34	−5.21, 2.53	0.50
Increase >10 points	302	1.33	1.37	−0.03	−2.44, 2.37	0.98
Total cholesterol: HDL-C						
All participants		−**0.08**	−**0.03**	−**0.06**	−**0.12, 0.01**	**0.10**
Median ΔHEI-2015						0.21^2^
Decrease >5 points	295	−0.11	−0.06	−0.05	−0.16, 0.07	0.44
No change	258	−0.04	−0.07	0.03	−0.10, 0.15	0.70
Increase 5–10 points	110	−0.11	−0.05	−0.07	−0.26, 0.12	0.49
Increase >10 points	301	−0.08	0.08	−0.16	−0.28, −0.04	0.09

Abbreviations: BMI, body mass index; HAT, Habitual Diet and Avocado Trial; HDL-C, high-density lipoprotein cholesterol; HEI, Healthy Eating Index.

1 Mixed models were used to assess the association between HEI-2015 change and intervention effects on cardiometabolic risk factors and make comparisons between intervention groups. The categorized change in the median total HEI-2015 score was included in the model along with an interaction term by intervention group.

2 *P* value is for the interaction term (median ΔHEI-2015 × intervention group).

## Discussion

In this study, a potentially clinically relevant increase in the HEI-2015 of 4.74 points was observed in response to provision of 1 avocado per day for 26 wk compared with continuing habitual intake in a large and diverse group of individuals with abdominal obesity. This improvement in the HEI-2015 was driven by greater intake of vegetables and more favorable fatty acid intake (higher intake of unsaturated fatty acids to saturated fatty acids), which directly reflects avocado consumption. The avocado-supplemented diet group also had improvements in the sodium, refined grains, and added sugars HEI-2015 components (reflecting lower intake) compared with the habitual diet group. No change in the saturated fats component was observed. These changes suggest avocados partially displaced foods containing sodium, added sugars, and refined grains, without influencing intake of foods containing saturated fatty acids. Some displacement of total protein and seafood and plant protein components also occurred with the avocado intervention, as reflected by the lower scores for these components. Although intake of 1 avocado per day resulted in an increase in adherence to the Dietary Guidelines for Americans, these improvements did not predict changes in risk factors for cardiometabolic disease over a 26-wk period.

Consistent with HEI-2015 estimates for the United States population, baseline scores in this cohort were in the poor range and slightly lower than the current mean United States estimate for individuals aged ≥20 y of 58 points [25]. Importantly, this single-food focused intervention resulted in an approximate 9% increase in the HEI-2015 score without significant heterogeneity across race, ethnicity, age, or BMI categories. The increase in the HEI-2015 was largely because of better scores for the fatty acids and total vegetable components. The increase in the fatty acids component score accounted for approximately half of the total improvement in the HEI-2015 score. As reported previously, the avocado-supplemented diet group had no change in saturated or polyunsaturated fatty acid intake compared with the habitual diet group [18]. However, intake of MUFAs was increased by 13 g/d (5.2% of total energy) in the avocado-supplemented diet group compared with the habitual diet group [18], which is close to the amount of monounsaturated fatty acids in the provided avocados (17 g). The increase in the total vegetable HEI-2015 component (0.99 points; 95% CI: 0.77, 1.21) in the avocado-supplemented diet group compared to the habitual diet group was less than the expected, given 1 avocado (168 g; 280 kcal) is equal to 1.16-cup equivalent of vegetables. However, a net increase in vegetable intake of ∼0.22-cup equivalents/1000 kcal was observed. These findings underscore the importance of assessing overall diet quality independent of food or diet quantity because diet is a complex, multidimensional behavior, and there are many ways to consume a given food.

The magnitude of improvement in the HEI-2015 score we observed was similar to that of previous clinical trials examining interventions focused on lifestyle management of diet-related chronic diseases with individual or group education (4–7 points) [14,15]. In this study, we extended previous work by examining diet quality change as a predictor of intervention effectiveness to improve cardiometabolic health. Overall, no heterogeneity in the intervention effects was observed by median change in the HEI-2015 score categorized by a decrease (>−5 points), no change (−5 to +5 points), or increase at 2 levels (5–10 points and >10 points) over the 26-wk follow-up period. These findings are somewhat surprising given the strong and consistent evidence showing higher diet quality [[6], [7], [8], [9], [10]] and improvements in diet quality over time [26,27] are associated with lower risk of incident cardiometabolic disease and mortality. However, it is possible that 26-wk of exposure to better diet quality is not long enough to detect improvements in cardiometabolic health. Observational investigations of the relationship between diet quality and cardiometabolic disease outcomes typically have follow-up periods that extend over multiple years, and improvements in diet quality are associated with relatively small changes in chronic disease risk factors. In an analysis of the Nurses’ Health Study I and II and the Health Professionals Follow-up Study, a 1-SD increase in the Alternative Healthy Eating Index-2010 (−0.47 kg; 95% CI: −0.67, −0.26 kg), the Alternate Mediterranean Diet score (−0.23 kg; 95% CI: −0.30, −0.16 kg), and the Dietary Approaches to Stop Hypertension score (−0.42 kg; 95% CI: −0.54, −0.29 kg) was associated with significantly less weight gain over 4 y. Similarly, in an analysis from the Multiethnic Cohort Study, per 1 SD (∼10 points) increase in the HEI-2015 score, body weight was reduced by 0.48 and 0.54 kg in males and females, respectively, over 10 y [28]. Therefore, it is likely that a greater follow-up period may be needed to detect small incremental improvements in cardiometabolic disease risk factors in response to improved diet quality.

It is also plausible that the increase in diet quality observed in this trial did not translate to an improvement in risk factors for cardiometabolic diseases because baseline HEI-2015 scores were so low. Despite, high adherence (88%–95% of the intervention group consumed at least some avocado on each recall day) and the 4.74-point improvement in the HEI-2015 observed with the avocado intervention, the HEI-2015 scores at follow-up remained in the lowest grade category (from an A to an F) receiving an F grade (0–59 points) [29]. We did see a trend, albeit not statistically significant, for participants who had greater than a 10-point increase (30%) in the HEI-2015 to have greater reductions in total cholesterol, LDL cholesterol, and the total cholesterol:high-density lipoprotein cholesterol ratio, which provides some evidence for the change in HEI-2015 that may be needed for blood cholesterol lowering. Further investigation of the improvement in diet quality needed to elicit clinically significant improvements in cardiometabolic disease risk factors is needed. This evidence will inform the development of programs, such as “food is medicine” interventions that both improve diet quality and diet-related risk factors for chronic disease.

An objective of these analyses was to examine how a single food-based intervention altered adherence to the Dietary Guidelines for Americans. To achieve this, the NDSR HEI-2015 calculation had to be modified to align with USDA’s classification of avocados as a vegetable. In the USDA’s Food Pattern Equivalents Database, avocados are considered a vegetable and, in the HEI-2015 calculation, only contribute to the total vegetables component (maximum score 5). Conversely, in the HEI-2015 code for NDSR created by the Nutrition Coordinating Center, avocados are considered a fruit and contribute to both the total fruits (maximum score 5) and whole fruits (maximum score 5) components [22]. However, this discrepancy in the NDSR HEI-2015 calculation resulted in an approximate 80% inflation of the HEI-2015 intervention effect estimate (NDSR HEI-2015 calculated total score 8.4 [18] compared with 4.74 points) in this study. However, baseline HEI-2015 scores for both groups and HEI-2015 scores at all time points for the habitual diet group remained unchanged [18]. This is because, in the habitual diet group, mean intake of avocados was ≤0.6 servings per day (0.5 cup or ∼75 g of chopped avocado = 1 serving) across all time points, and 92%–94% of participants did not consume avocado. In the avocado-supplemented group, mean intake of avocado was 0.7 servings per day at baseline, with 91% of participants consuming no avocado. In this group, mean avocado intake increased to ∼2 servings per day at the follow-up time points, with 88%–95% of participant consuming some avocado. Collectively, these findings suggest that avocados classification as either a fruit or vegetable only affects HEI-2015 calculation when a large proportion of a sample is consuming up to 1 avocado per day. Data from NHANES 2001–2012 suggest only 2% of United States adults are avocado consumers [19,20], and therefore, this issue is only likely to meaningfully affect HEI-2015 scores in certain situations where a large proportion of a cohort is consuming avocado.

Strengths of this study include the use of a large and diverse sample from a 26-wk multicenter randomized controlled trial conducted in 4 locations across the United States. In addition, 24-h recalls were conducted at baseline and 3 time points after randomization, which is consistent with diet assessment recommendations for examining the effect of an intervention on mean usual intake [30]. However, we acknowledge that the National Cancer Institute (Markov Chain Monte Carlo) multivariate algorithm [31] was not used to estimate the effect of HEI-2015 change on the change in cardiometabolic disease risk factors, and therefore, our results may be influenced by random measurement error that could bias the results in any direction [32,33]. This work is also limited by the use of only 1 diet quality index. Although evidence from the National Institutes of Health-National Cancer Institute Dietary Patterns Methods Project shows that HEI rankings are consistent with other diet quality indices such as the Alternative Healthy Eating Index, the Alternate Mediterranean Diet score, and the Dietary Approaches to Stop Hypertension score [34]. In addition, higher scores for all these indices are associated with similar reductions in risk of all-cause, cardiovascular-related, and cancer-related mortality. Finally, we examined dietary incorporation of avocados, a vegetable, in these analyses, and given the unique fatty acid profile of avocados, these results are not generalizable to other vegetables.

Intake of 1 avocado per day as part of usual intake for 26 wk in free-living adults with abdominal obesity increased adherence to the Dietary Guidelines for Americans. However, changes in the HEI-2015 over the 26-wk period did not predict changes in risk factors for cardiometabolic disease. Further research is needed to determine the magnitude of diet quality improvement needed to improve cardiometabolic disease risk factors with consideration for baseline diet quality.

## Author contributions

The authors’ responsibilities were as follows – KSP, SS, AHL, NM, ZL, JS, DMR, PMK-E: designed the research; SS: conducted the statistical analyses; KSP, SS: drafted the manuscript; and all authors: critically reviewed and accepted the final version of the manuscript.

## Conflict of interest

The authors report no conflict of interest.

## Funding

This work was support by the Avocado Nutrition Center. The funder had no role in the data analysis, data interpretation, or writing of the manuscript.

## Data availability

Data described in the manuscript, code book, and analytic code will be made available on request pending application and approval.
