# Supplementary material for: One Avocado per Day as Part of Usual Intake Improves Diet Quality: Exploratory Results from a Randomized Controlled Trial
Source: Curr Dev Nutr. 2024 Jan 11;8(2):102079. doi: 10.1016/j.cdnut.2024.102079 (PMC10875193; doi:10.1016/j.cdnut.2024.102079)
Supplement: Supplementary file 1 [file mmc1.docx]

**Supplementary Material for Online Publication**

## Supplementary Table 1: Model-based estimates of the between group difference in changes in HEI-2015 total and component scores during the 26-week HAT adjusted for baseline HEI-2015 score^1^

| **Variable** | **Avocado Supplemented Diet Group** | | | **Habitual Diet Group** | | | **Estimated Mean Difference**  **(95% CI)** | **P-value** |
| --- | --- | --- | --- | --- | --- | --- | --- | --- |
|  | Wk. 8  n=490 | Wk. 16  n=479 | Wk. 26  n=472 | Wk. 8  n=486 | Wk. 16  n=487 | Wk. 26  n=480 |  |  |
| HEI-2015 Score^2^ | 3.95 | 3.36 | 3.50 | −1.07 | −0.96 | −0.18 | 4.34 (3.03, 5.65) | <0.001 |
| Total Vegetables^3^ | 1.76 | 1.75 | 1.70 | 0.70 | 0.62 | 0.78 | 1.04 (0.91, 1.17) | <0.001 |
| Greens & Beans^3^ | −0.31 | −0.34 | −0.32 | −0.16 | −0.21 | −0.25 | −0.12 (−0.32, 0.08) | 0.24 |
| Total Fruit^3^ | −0.70 | −0.57 | −0.58 | −0.52 | −0.45 | −0.46 | −0.14 (−0.31, 0.03) | 0.11 |
| Whole Fruit^3^ | −0.52 | −0.46 | −0.36 | −0.41 | −0.29 | −0.27 | −0.13 (−0.32, 0.07) | 0.20 |
| Whole Grains^4^ | −0.90 | −1.10 | −0.92 | −0.98 | −0.97 | −0.85 | −0.04 (−0.38, 0.30) | 0.83 |
| Dairy^4^ | −0.61 | −0.32 | −0.72 | −0.20 | −0.18 | −0.16 | −0.38 (−0.69, −0.07) | 0.02 |
| Total Protein Foods^3^ | 1.18 | 1.19 | 1.25 | 1.58 | 1.51 | 1.51 | −0.33 (−0.45, −0.22) | <0.001 |
| Seafood & Plant Protein^3^ | −0.23 | −0.19 | −0.39 | 0.02 | −0.03 | 0.11 | −0.31 (−0.50, −0.11) | 0.002 |
| Fatty Acid Ratio^4^ | 2.33 | 2.10 | 2.23 | −0.15 | −0.12 | −0.14 | 2.36 (2.04, 2.67) | <0.001 |
| Sodium^4^ | 0.79 | 0.29 | 0.66 | −0.71 | −0.59 | −0.27 | 1.11 (0.78, 1.44) | <0.001 |
| Refined Grains^4^ | 1.97 | 1.91 | 1.93 | 1.34 | 1.22 | 1.33 | 0.64 (0.33, 0.94) | <0.001 |
| Added Sugars^4^ | 2.47 | 2.58 | 2.42 | 1.92 | 1.93 | 2.06 | 0.52 (0.28, 0.77) | <0.001 |
| Saturated Fats^4^ | 0.26 | 0.03 | 0.14 | −0.02 | 0.05 | −0.09 | 0.16 (−0.17, 0.49) | 0.33 |

^1^Mixed models were used to estimate the difference in the change in total HEI-2015 score and HEI-2015 component scores from baseline by randomization with adjustment for study site and baseline score as a categorical variable. HAT, Habitual Diet and Avocado Trial; HEI-2015, Healthy Eating Index-2015

^2^maximum score 100

^3^maximum score 5

^4^maximum score 10

## Supplementary Table 2: Model-based estimates of change in total HEI-2015 score in subgroups by intervention group at 26-weeks in HAT adjusted for baseline HEI-2015 value

| **Subgroups** | **n** | **Avocado Supplemented Diet Group** | **Habitual Diet Group** | **Estimated Between-Group Difference in Change** | | | |
| --- | --- | --- | --- | --- | --- | --- | --- |
|  |  |  |  | **Mean** | **P-value** | **95% CI** | **P-value^2^** |
| **Sex** |  |  |  |  |  |  | 0.16 |
| Women | 730 | 5.12 | 0.11 | 5.01 | <0.001 | 3.48, 6.54 |  |
| Men | 278 | 2.78 | −0.14 | 2.92 | 0.02 | 0.41, 5.43 |  |
| **Self-reported ethnicity** |  |  |  |  |  |  | 0.21 |
| Not Hispanic/Latino | 797 | 4.67 | −0.17 | 4.84 | <0.001 | 3.38, 6.31 |  |
| Hispanic/Latino | 207 | 3.58 | 0.83 | 2.75 | 0.06 | −0.14, 5.65 |  |
| **Self-reported race** |  |  |  |  |  |  | 0.31 |
| Black | 149 | 5.54 | −0.95 | 6.49 | 0.0002 | 3.07, 9.91 |  |
| Asian | 58 | 6.37 | 0.00 | 6.36 | 0.02 | 0.94, 11.79 |  |
| White | 694 | 4.29 | −0.01 | 4.30 | <0.001 | 2.72, 5.87 |  |
| Other | 104 | 3.36 | 1.68 | 1.68 | 0.42 | −2.45, 5.82 |  |
| **BMI** |  |  |  |  |  |  | 0.42 |
| Healthy/ Overweight | 317 | 4.89 | 1.48 | 3.42 | 0.004 | 1.11, 5.73 |  |
| Obese Class I | 386 | 4.72 | −0.14 | 4.86 | <0.001 | 2.75, 6.97 |  |
| Obese Class II | 192 | 3.85 | 0.33 | 3.52 | 0.02 | 0.53, 6.51 |  |
| Obese Class III | 112 | 2.83 | −4.17 | 7.01 | 0.0005 | 3.04, 10.97 |  |
| **Age Group** |  |  |  |  |  |  | 0.16 |
| 25-30 y | 96 | 2.37 | 0.69 | 1.67 | 0.45 | −2.65, 6.00 |  |
| 31-59 y | 644 | 4.29 | −1.03 | 5.32 | <0.001 | 3.69, 6.95 |  |
| 60+ y | 268 | 5.54 | 2.36 | 3.17 | 0.01 | 0.68, 5.66 |  |
| **Site** |  |  |  |  |  |  | 0.19 |
| LLU | 251 | 5.00 | −0.98 | 5.98 | <0.001 | 3.39, 8.58 |  |
| UCLA | 251 | 5.56 | 1.99 | 3.57 | 0.008 | 0.94, 6.20 |  |
| Penn State – UP | 134 | 1.59 | −0.33 | 1.92 | 0.29 | −1.65, 5.49 |  |
| Penn State – Hershey | 119 | 3.95 | 1.60 | 2.35 | 0.23 | −1.45, 6.15 |  |
| Tufts | 253 | 5.11 | −0.81 | 5.91 | <0.001 | 3.31, 8.52 |  |

^1^Mixed models were used to estimate the difference in the change in total HEI-2015 score from baseline by randomization and subgroup with adjustment for study site baseline score as a categorical variable. The p-values from the interaction term of subgroup and randomization were used to test the significance of subgroup effects. BMI, body mass index; HAT, Habitual Diet and Avocado Trial; HEI-2015, Healthy Eating Index-2015; LLU, Loma Linda University; UCLA, University of California at Los Angeles; Penn State – UP, Penn State University - University Park Campus; Penn State – Hershey, Penn State College of Medicine, Hershey; Tufts, Tufts University

^2^P value for difference between estimates within a subgroup

## Supplementary Table 3: Avocado intake by intervention group during the 26-weeks of HAT^1^

| Avocado Consumption | **Avocado Supplemented Diet Group** | | | | **Habitual Diet Group** | | | |
| --- | --- | --- | --- | --- | --- | --- | --- | --- |
|  | Baseline | Wk. 8 | Wk. 16 | Wk. 26 | Baseline | Wk. 8 | Wk. 16 | Wk. 26 |
|  | n=505 | n=490 | n=479 | n=472 | n=503 | n=486 | n=487 | n=480 |
| None | 459 (90.89) | 24 (4.9) | 39 (8.14) | 56 (11.86) | 461 (91.65) | 458 (94.24) | 452 (92.81) | 439 (91.46) |
| Some^2^ | 41 (8.12) | 82 (16.73) | 75 (15.66) | 57 (12.08) | 41 (8.15) | 27 (5.56) | 35 (7.19) | 40 (8.33) |
| One+/day | 5 (0.99) | 384 (78.37) | 365 (76.2) | 359 (76.06) | 1 (0.2) | 1 (0.21) | 0 (0.0) | 1 (0.21) |

^1^Data presented as n (%); Data derived from 24-hour diet recalls collected for specified week by intervention group. HAT, Habitual Diet and Avocado Trial

^2^Some defined as avocado consumption reported in the recall that was <90% of the study-provided amount of 168 g (151 g) i.e., total avocado intake <151 g; avocado reported but no amount given; avocado on toast, bread, salad, sandwich but no amount given and no note for amount, and guacamole reported in the recall that was less than the Nutrition Data System for Research equivalent to 168 g of avocado (1.5 cups); total guacamole <1.5 cups; and guacamole reported but no amount given.
